# Biochar in the Remediation of Organic Pollutants in Water: A Review of Polycyclic Aromatic Hydrocarbon and Pesticide Removal

**DOI:** 10.3390/nano15010026

**Published:** 2024-12-27

**Authors:** Jelena Beljin, Nina Đukanović, Jasmina Anojčić, Tajana Simetić, Tamara Apostolović, Sanja Mutić, Snežana Maletić

**Affiliations:** Faculty of Sciences, University of Novi Sad, 21000 Novi Sad, Serbia; nina.djukanovic@dh.uns.ac.rs (N.Đ.); jasmina.anojcic@dh.uns.ac.rs (J.A.); tamara.apostolovic@dh.uns.ac.rs (T.A.); sanja.mutic@dh.uns.ac.rs (S.M.); snezana.maletic@dh.uns.ac.rs (S.M.)

**Keywords:** biochar, PAH, pesticide, water, wastewater treatment

## Abstract

This review explores biochar’s potential as a sustainable and cost-effective solution for remediating organic pollutants, particularly polycyclic aromatic hydrocarbons (PAHs) and pesticides, in water. Biochar, a carbon-rich material produced from biomass pyrolysis, has demonstrated adsorption efficiencies exceeding 90% under optimal conditions, depending on the feedstock type, pyrolysis temperature, and functionalization. High surface area (up to 1500 m^2^/g), porosity, and modifiable surface functional groups make biochar effective in adsorbing a wide range of contaminants, including toxic metals, organic pollutants, and nutrients. Recent advancements in biochar production, such as chemical activation and post-treatment modifications, have enhanced adsorption capacities, with engineered biochar achieving superior performance in treating industrial, municipal, and agricultural effluents. However, scaling up biochar applications from laboratory research to field-scale wastewater treatment poses significant challenges. These include inconsistencies in adsorption performance under variable environmental conditions, the high cost of large-scale biochar production, logistical challenges in handling and deploying biochar at scale, and the need for integration with existing treatment systems. Such challenges impact the practical implementation of biochar-based remediation technologies, requiring further investigation into cost-effective production methods, long-term performance assessments, and field-level optimization strategies. This review underscores the importance of addressing these barriers and highlights biochar’s potential to offer a sustainable, environmentally friendly, and economically viable solution for large-scale wastewater treatment.

## 1. Introduction

The contamination of soil, sediments, and water with both organic and inorganic pollutants have become an issue of global concern due to its far-reaching impacts on environmental health and ecosystem stability [1,2]. Organic pollutants, particularly polycyclic aromatic hydrocarbons (PAHs) and pesticides, originate from a wide range of human activities, including industrial processes, agricultural applications, and the improper disposal of waste materials [3]. These compounds are characterized by their persistence, bioaccumulation potential, and toxic effects, which make them especially problematic for both aquatic and terrestrial ecosystems [4,5]. Due to their chemical stability and resistance to degradation [6], organic pollutants remain in the environment for extended periods, increasing the risk of exposure to plants, animals, and humans. This persistence also leads to the bioaccumulation and biomagnification of pollutants through food chains, where they can reach toxic concentrations in higher trophic levels [7,8].

PAHs, which are hazardous colorless or pale compounds with two or more benzene rings and various structural configurations, have become globally widespread due to their biological and pyrogenic origins. Their chemical stability and limited biological availability make PAHs persistent in the environment. They are generally classified as low- and high-molecular-weight PAHs based on molecular composition. The physical and chemical properties of PAHs are influenced by their molecular weight and functional group content. Due to their persistence, as well as carcinogenic and mutagenic effects, PAHs are recognized as significant pollutants, and their stability and accumulation tendencies contribute to their wide distribution in nature [9,10].

Pesticides are frequently detected in surface freshwater as complex mixtures, usually at concentrations below 1000 μg/L [11]. Various methods exist for removing and degrading pesticides [12]. However, there is growing interest in the removal of water pollutants, including pesticides, through adsorption onto efficient biodegradable materials due to their simplicity and practicality [13]. Atrazine, chlorpyrifos, chlorfenvinphos, cyprodinil, diazinon, dimethoate, diuron, ethion, malathion, and profenofos were selected for this study because of their extensive use, persistence, and frequent detection in previous research [11]. Additionally, the European Water Framework Directive (WFD) 2000/60/EC [14] lists 33 priority substances, including atrazine, diuron, and chlorfenvinphos, which are particularly concerning due to their environmental mobility and potential risks to human health and aquatic ecosystems.

Conventional approaches to remediate these contaminants, such as chemical treatments, physical filtration, and biological degradation, are often inadequate, particularly when applied to complex, real-world environmental matrices where contaminants are mixed with high concentrations of background electrolytes and other co-occurring substances [15]. This challenge has prompted the exploration of biochar as a viable, cost-effective, and environmentally sustainable alternative for remediation. Biochar, a carbon-rich byproduct of biomass pyrolysis, is produced from diverse feedstocks, including coconut shells, straw, wood, and other organic waste materials. Its properties, such as high surface area, porosity, and functional groups, make it an effective adsorbent for removing contaminants from water and soil [1,16].

However, while pristine biochar has shown promise in laboratory settings, it frequently falls short in field applications due to limitations in its adsorption efficiency, selectivity, and overall stability under varying environmental conditions. Biochar’s performance can be negatively affected by factors such as pH variations, competing ions, and the presence of organic matter that can reduce its capacity to bind with target pollutants [17]. Consequently, there is an increasing focus on developing engineered or modified biochar with enhanced properties tailored to specific environmental applications. Engineered biochar, created by incorporating functional groups or doping with specific elements such as sulfur, nitrogen, or metals, offers improved adsorption capacity, selectivity, and catalytic activity, making it a more effective solution for contaminant remediation [18]. These modifications can involve adjusting pyrolysis parameters, adding chemical reagents during production, or incorporating additives that enhance biochar’s reactivity and surface properties.

The application of engineered biochar in field settings, however, remains limited, with most research confined to controlled laboratory experiments. Field applications are complicated by environmental heterogeneity, where factors like fluctuating temperatures, seasonal wetting and drying cycles, and soil type variability can impact biochar’s performance [19,20,21]. Understanding the long-term interactions between engineered biochar and environmental matrices is crucial for developing effective remediation strategies. Furthermore, as access to clean water becomes increasingly essential due to population growth and industrial development, innovative and cost-effective solutions for water remediation are urgently needed. The unique properties of biochar, combined with its relatively low production cost, offer a promising approach to water decontamination that could help alleviate pollution challenges, particularly in areas with limited access to advanced water treatment technologies. In addressing the limitations of conventional water treatment methods, such as high costs and complex operational requirements, biochar-based technologies represent a sustainable and scalable solution that could bridge the gap between laboratory successes and field applications [3,22].

Identifying gaps in the use of biochar for the remediation of organic pollutants, particularly PAHs and pesticides, reveals several critical areas for further research. While biochar demonstrates potential for pollutant removal, limitations and knowledge gaps persist. This review aims to identify the main problems and shortcomings identified in published papers so far to overcome these problems. The usage of biochar is well explained in the literature while the field of water remediation and organic pollutants removal remains insufficiently researched. This review examines the sustainability and cost-effectiveness of both modified and unmodified biochar as essential factors in assessing its potential for pollutant removal of PAHs and pesticides from water.

## 2. Production of Biochar and Application for PAH and Pesticide Removal from Water

**Figure 1:**
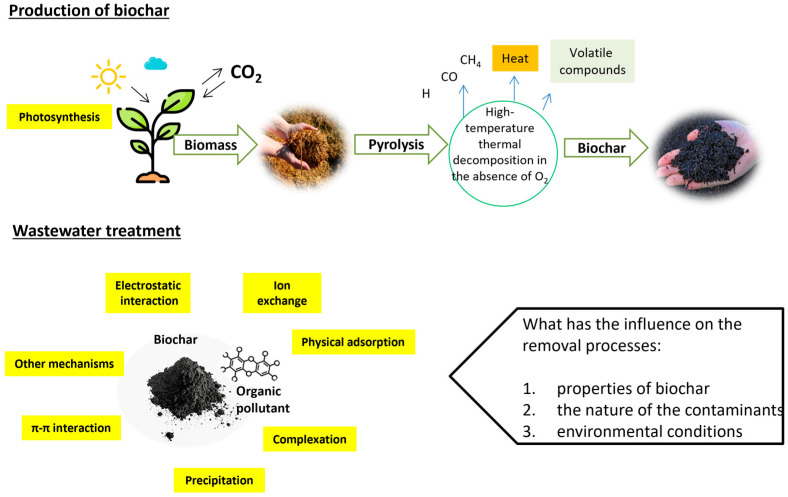
Biochar production and factors that influence removal mechanisms.

The adsorption mechanisms of PAHs and pesticides on biochar are influenced by several key factors, including the physicochemical properties of biochar, the nature of the contaminants, and environmental conditions (Figure 1). Understanding these factors is crucial for optimizing biochar’s effectiveness in remediation efforts. For example, some physicochemical properties of biochar, like surface area and pore structure, higher surface area, and microporosity, enhance adsorption capacity, particularly for organic contaminants like PAHs [23,24], while the presence of various functional groups on biochar can facilitate interactions through hydrogen bonding and electrostatic attraction, improving sorption efficiency [24].

Furthermore, contaminant characteristics like the number of aromatic rings in PAHs affect their adsorption; more complex structures may require specific biochar types for effective remediation [25]. Also, hydrophobic interactions play a significant role in the adsorption of organic pollutants, with biochar’s hydrophobic surfaces enhancing the retention of these compounds [23]. Environmental conditions like pH and ionic strength can alter the charge of both biochar and contaminants, influencing adsorption dynamics [26]. Finally, biochar modifications such as phosphoric acid modification can significantly enhance biochar’s adsorption capacity for specific contaminants like pesticides [27,28,29]. Biochar can be produced from a range of waste materials originating from agricultural biomass, industrial processes, municipal sewage, and biological sources [30]. Table 1 lists the different types of feedstocks used in biochar production, production conditions, physico-chemical differences in gained material, and removal efficiency reported in the literature.

The efficiencies of different biochar in removing PAHs and pesticides from wastewater vary significantly based on their feedstock source and preparation conditions (Table 1). Biochar derived from agricultural residues, forestry waste, and municipal solid waste exhibits different adsorption efficiencies due to variations in surface functional groups and elemental composition [31]. Modifying biochar through activation or composite formation enhances its ability to remove persistent organic pollutants (POPs) like pesticides and pharmaceuticals [32]. Biochar-based catalysts in advanced treatment technologies, such as photocatalysis and electro-Fenton processes, have shown increased efficacy in degrading POPs compared to conventional methods [33].

**Table 1:** Biomass types used for water remediation and their properties.

Biomass Types	Carbon Content (%)	Ash Content (%)	Porosity (%)	Surface Area (m^2^/g)	Targeted Pollutant	Removal Efficiency (%)	Production Conditions	Reference
Casuarina	40–55	2–3	68–76	200–350	Naphtalen	63	25 °C to 900 °C; various heating rates (2.5, 5, and 15 °C min^−1^), followed by nitrogen purging at a rate of 40 mL min^−1^.	Nama et al. [34]
Pine sawdust	55–65	2–6	2–6	180–380	polystyrene microplastics	84.8–96.2	The Fe-impregnated pretreated pine sawdust, Fe/Mg modified biochar (Fe/MgBC), and Fe/ZnBC were pyrolyzed at 550 °C for 2 h in a tube furnace with a heating rate of 5 °C/min.	Zhang et al. [35]
Sugarcane bagasse 300	48.16	4.23	/	224.07	2,4,6-Trichlorophenol 2,4,6-Trichlorophenol 2,4,6-Trichlorophenol	9.21–9.97	The dried Sugarcane bagasse was charred in a muffle furnace at different temperatures (573, 673, and 773 K) for 20 min.	Mubarik et al. [36]
Sugarcane bagasse 400	50.47	4.17	/	361.77				
Sugarcane bagasse 500	49.97	4.08 ± 0.21	/	291.37				
Maize straw 350	58.9	15.4	/	6.71	Carbaryl	61.1–79.5	The materials were pyrolyzed at 350 or 700 °C for 2 h in a closed container under an oxygen-limited condition in a preheated muffle furnace.	Ren et al. [37]
Maize straw 700	43.8	21.9	/	265		56.9–67.1		
Pig manure 350	31.6	45.3	/	23.8		60–75		
Pig manure 700	25.2	66.8	/	32.6		65.7–78.6		
Rice straw 350	44.5	29.1	/	9.01		71.8–73.2		
Rice straw 700	56.7	38.2	/	188		58.3–72.6		
Pine wood	85.2	1.8	/	/	Phenanthrene	<15	Pyrolysis at 450 °C (15 min).	Jiménez et al. [38]
Olive pruning	82.9	10						
Rice husk	43.1	52						
Sewage sludge 400	32.7	53.5		11.4	Phenol	≤12.6	Pyrolysis at 400 and 700 °C for 4 h.	Liang et al. [39]
Sewage sludge 700	31.9	61.3		63.1		≤12.6		
Sewage sludge 400—demineralized	47	22.3		10.6		36.3		
Sewage sludge 700—demineralized	51.5	30.0		219		57.8		
Willow				11.4 (840.6)	PAHs	41–84	Pyrolysis at 350–650 °C.	Kotlowski et al. [40]
Coconut				3.1 (626.8)				
Wheat straw				26.3 (246.2)				

The yield and stability of biochar are significantly influenced by both pyrolysis temperature and the type of feedstock used. Research indicates that higher pyrolysis temperatures generally lead to lower biochar yields but enhance its carbon content and stability. The characteristics of the feedstock also play a crucial role, as different materials yield biochar with varying properties. As pyrolysis temperature increases from 400 °C to 800 °C, biochar yield decreases due to enhanced volatilization of organic compounds [41]. Higher temperatures (≥700 °C) result in biochar with greater chemical stability, making them more suitable for long-term carbon storage [42,43], and increased temperature enhances the biochar’s bulk density, surface area, and thermal stability [41].

Different feedstocks, such as rosemary leaves and stems, exhibit distinct chemical compositions, affecting biochar properties. For instance, stem-derived biochar have higher carbon stability compared to those from leaves [43]. Feedstocks with higher nitrogen content, like chicken manure, retain more nitrogen in the resulting biochar when pyrolyzed at lower temperatures [44]. Thus, a balance between temperature and feedstock type is essential for optimizing biochar production for specific applications.

## 3. Mechanisms of PAH and Pesticide Removal

The increasing contamination of water by persistent organic pollutants such as PAHs and pesticides has driven the development of innovative and sustainable treatment methods. Among these, biochar has garnered attention for its efficiency in absorbing and degrading such pollutants. However, optimizing biochar’s performance requires a deep understanding of its interactions with PAHs and pesticides [45,46,47]. This section explores these interactions, emphasizing the critical factors that govern biochar’s efficiency in water treatment. The removal of polycyclic aromatic hydrocarbons (PAHs) and pesticides from water is essential due to their persistence, toxicity, and potential to bioaccumulate, posing significant environmental and health risks. Various physical, chemical, and biological mechanisms have been developed to address these contaminants effectively. Physical methods, such as adsorption onto biochar, rely on the affinity of PAH and pesticide molecules to stick to adsorbent surfaces, allowing separation from water. Biological processes also contribute significantly, as certain microorganisms can metabolize PAHs and pesticides, converting them into simpler, non-toxic compounds. Each method has its advantages and limitations, and often, a combination of these approaches is used to achieve optimal removal efficiency in water treatment systems.

One of the most important aspects of biochar’s interaction with PAHs and pesticides is its surface characteristics. These include surface area, porosity, and the presence of functional groups, all of which influence the adsorption process. Biochar with a high proportion of micropores (<2 nm) and mesopores (2–50 nm) offers a larger surface area for the adsorption of hydrophobic pollutants like PAHs and certain pesticides [18,48,49]. These pores provide physical sites where the pollutants can be trapped. Oxygen-containing functional groups, such as hydroxyl, carboxyl, and carbonyl, can enhance the adsorption of polar pesticides through hydrogen bonding and electrostatic interactions [29].

The optimization of these properties through controlled pyrolysis and post-treatment modifications (e.g., chemical activation or functionalization) is critical for maximizing biochar’s efficacy [50,51]. To optimize biochar for the removal of PAHs and pesticides, various strategies can be employed:

1. Chemical Activation: Impregnation of biochar with acids, bases, or metals can enhance porosity and introduce functional groups, increasing affinity for specific pollutants [50,51]. Chemical methods, particularly advanced oxidation processes (AOPs) like ozonation and UV/H₂O₂ treatment, utilize highly reactive species, such as hydroxyl radicals, to break down complex molecules into less harmful or more biodegradable forms [2].
2. Magnetic Modification: Incorporating magnetic nanoparticles into biochar facilitates easy recovery after use, making it more practical for large-scale applications [52,53].
3. Multi-Functionality: Designing biochar capable of simultaneous adsorption and degradation of pollutants, such as by incorporating photocatalytic materials, can improve treatment outcomes [54,55].

**Figure 2:**
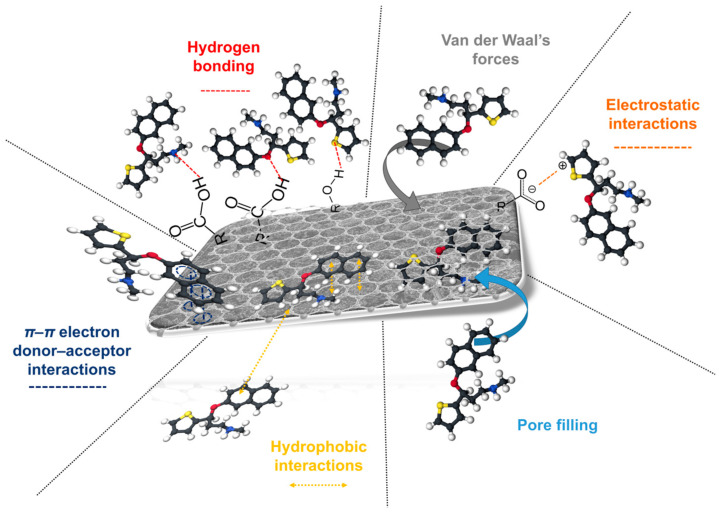
Interaction responsible for PAH and pesticide removal.

Biochar’s interactions with PAHs and pesticides are complex and often influenced by synergies between adsorption mechanisms. For example, hydrophobic and electrostatic interactions can act simultaneously, leading to high removal efficiencies. However, challenges such as competition with co-pollutants and biochar fouling remain significant hurdles. Figure 2 and Table 2 give a review of interactions that are responsible for PAH and pesticide removal from water samples. Biochar can facilitate chemical reactions that degrade PAHs, particularly when combined with reactive agents like peroxymonosulfate (PMS) [56]. The immobilization of bacteria on biochar enhances the biodegradation of PAHs, as seen with chlorpyrifos degradation [57].

PAHs are highly toxic pollutants that persist in the environment due to their resistance to degradation. They pose significant threats to aquatic ecosystems and human health. Effective removal techniques (Table 3) are critical to addressing their presence in wastewater [58,59]. Among the methods studied, biochar-based techniques have emerged as promising solutions due to their efficacy, sustainability, and cost-effectiveness [60]. This group of pollutants is characterized by their hydrophobic nature and aromatic ring structures, which interact with biochar predominantly through hydrophobic interactions and π-π stacking [61,62]. Biochar with nonpolar surfaces, often produced at higher pyrolysis temperatures, exhibit enhanced affinity for PAHs [48]. The removal efficiency is closely tied to the hydrophobicity of both the biochar surface and the PAHs [61]. The graphitic domains in biochar, formed during pyrolysis, facilitate π-π interactions with the aromatic rings of PAHs, further enhancing adsorption. These mechanisms highlight the need for biochar with high aromaticity and low polarity to maximize PAH removal.

The effectiveness of biochar in removing PAHs from contaminated water has been extensively studied, revealing its potential as a sustainable remediation strategy. Biochar’s unique properties, such as high surface area and functional groups, enhance its adsorption capabilities, making it a viable option for PAH removal. Biochar effectively absorbs PAHs due to its porous structure and large surface area, leading to significant reductions in PAH concentrations in water [20]. Yaashikaa et al. [24] demonstrate significant effectiveness in removing PAHs from contaminated water due to their large surface area, abundant functional groups, and pore structure, which enhance adsorption and facilitate the remediation of these toxic pollutants. On the other hand, composite materials, such as biochar loaded with humic acid, can activate persulfate, resulting in enhanced degradation of PAHs into less toxic intermediates [63]. Ball milling biochar to nanoscale increases its surface area and reactivity, leading to a 1.1 to 2.9-fold decrease in freely available PAHs [64]. The type of biomass and pyrolysis temperature significantly affect biochar’s adsorption capacity and efficiency in PAH removal (physicochemical properties and performance of non-woody derived biochar for the sustainable removal of aquatic pollutants: a systematic review). These differences are presented in Table 1. Variations in biochar’s surface characteristics, such as hydrophilicity and pore volume, influence its interaction with PAHs [19,20,64].

**Table 2:** Interactions and proposed mechanisms in the literature.

References	Interactions	Investigated Problem/Solution/Result
Yao et al. [65]	The mechanisms involved in pesticide removal using KOH-activated biochar include van der Waals forces, pore filling, hydrogen bonding, and π-π electron donor–acceptor interactions, indicating a complex interaction during the adsorption process for effective contaminant removal.	KOH-activated biochar effectively removes acetamiprid and triadimefon. The maximum adsorption capacity for acetamiprid is 40.41 mg g^−1^.
Chen et al. [56]	This paper discusses the removal of pesticides, specifically atrazine, using a green biochar iron composite (PC-ZVI) activated by peroxymonosulfate (PMS) and oxalic acid (OA). Mechanisms include reduction, oxidation, hydroxylation, substitution, and de-alkylation processes for effective degradation.	PMS enhances Cr(VI) and atrazine removal efficiency. PC-ZVI shows high recyclability and low toxicity.
Koippully Manikandan et al. [57]	This paper focuses on chlorpyrifos (CP) removal, highlighting a synergistic mechanism involving bacterial metabolism and adsorption. The immobilized Aeromonas veronii on rice husk biochar enhances pesticide degradation, effectively removing contaminants from water through these combined processes.	96.25% chlorpyrifos removal in water within 24 h. 92.4% removal in soil within 42 days.
Eissa et al. [66]	The paper reveals that active adsorption groups and metal oxides in biochar play a significant role in pesticide sorption.	Rise husk biochar showed the highest pesticide removal percentages among biochar. Adsorption equilibrium achieved in 60–120 min for different biochar.
Cao et al. [67]	The mechanisms involved in pesticide removal using boric acid-modified walnut shell biochar (WAB4) include hydrogen bonding, pore filling, hydrophobic effects, and π–π interactions, contributing to its high adsorption capacity for various pesticides in water.	WAB4 effectively absorbs investigated pesticides over 70%. No significant acute toxicity to Daphnia magna.
Matos et al. [68]	This paper discusses adsorption as the primary mechanism for pesticide removal from water using biochar. The activated and magnetized biochar effectively adsorbed thiacloprid and thiamethoxam, with a pseudo-second-order model describing the kinetics of adsorption.	Activated biochar absorbs 1.02 mg thiacloprid and 0.97 mg thiamethoxam. Magnetized biochar adsorbs 0.73 mg thiacloprid and 0.40 mg thiamethoxam.
Yaashikaa et al. [24]	This review discusses various mechanisms for PAH removal using biochar, including adsorption due to its large surface area and functional groups, as well as surface modification methods like magnetization, which enhance biochar’s effectiveness in removing pollutants from water.	Biochar effectively remediates polycyclic aromatic hydrocarbons (PAHs). Surface modifications enhance biochar’s properties for pollutant removal.
Murtaza et al. [69]	The mechanisms involved in the removal of polycyclic aromatic hydrocarbons (PAHs) and pesticides from water using biochar include electrostatic interaction, partitioning, hydrophobic interaction, and pore filling, which enhance the adsorption efficiency of these organic pollutants.	Engineered biochar composites have a high adsorption capacity for removing aquatic pollutants. Clay-based biochar composites are efficient in removing antibiotics, dyes, metals, and nutrients.

While biochar demonstrates considerable promise in PAH remediation, challenges such as varying PAH structures and environmental conditions can affect its overall effectiveness. Ke et al. [63] pointed out that biochar loaded with humic acid demonstrated high effectiveness in removing PAH, achieving removal efficiencies of 98.2% for naphthalene, 99.3% for pyrene, and 90.1% for benzo(a)pyrene from aqueous solutions through synergistic adsorption and persulfate activation. This paper also emphasizes that there are limitation factors like pH fluctuation, which inhibited remediation efficiency, and interfering ions, which reduced overall effectiveness. Raczkiewicz et al. [64] found that nanoscale biochar showed reduced effectiveness in immobilizing PAHs, with log K_d_ values decreased by 14 to 41% compared to bulk biochar, indicating a diminished capacity for PAH removal from contaminated water.

Some researchers deal with modified biochar materials. Li et al. [54] indicate that biochar significantly enhances the biodegradation of PAHs in contaminated water, especially when combined with a TiO2@biochar composite under light irradiation, improving the overall effectiveness of PAH removal through photocatalytic mechanisms. Also, they pointed out the limitation of this kind of process, that high dosages of biochar and TiO2@biochar inhibit cell growth and damage cell walls. Kang et al. [70] indicated that iron-modified biochar significantly enhances the removal of benzofluoranthrene in constructed wetlands, with effectiveness mediated by dissolved organic matter and microbial responses.

**Table 3:** Different methods used for PAH removal and their proposed mechanisms.

Method	Mechanism	Efficiency	Key Notes	References
Biochar adsorption	π–π interactions, hydrophobic interactions, and pore filling	Up to 81% (100 mg/L, 120 min)	Effective for various PAHs, low-cost	Li et al. [71] Kong et al. [60]
AOPs	ROS-mediated oxidation (e.g., -OH, -O_2_^−^)	Over 90% (enhanced with biochar)	Sustainable, broad-spectrum removal	Miklos et al. [72]
Photocatalysis	UV-activated TiO_2_	Up to 94.5% (5:1 biochar: TiO_2_ ratio)	Efficient for high-molecular PAHs	Boffito et al. [73]

Pesticides also vary significantly in their chemical structure, leading to diverse interaction mechanisms with biochar [28]. For nonpolar or weakly polar pesticides, such as organochlorine and organophosphorus compounds, hydrophobic interactions dominate [74]. Like PAHs, biochar’s aromaticity and porosity are crucial. Pesticides with polar functional groups, such as glyphosate or atrazine, can form electrostatic interactions with charged biochar surfaces [75]. Adjusting biochar’s surface charge through modification can optimize this interaction. Functionalized biochar, with groups like amines or carboxyls, can form hydrogen bonds or coordinate bonds with polar pesticides, improving adsorption capacity. Understanding the chemical structure of target pesticides is therefore essential for designing biochar with specific functional properties [76,77]. In the literature, the authors reported that weak interactions contribute to the binding of pesticides to biochar surfaces [65,78,79] and that the presence of functional groups on biochar enhances hydrogen bonding with pesticide molecules [65]. Also, it has been reported that the porous structure of biochar allows pesticides to be trapped within its matrix [66], and π-π Interactions allow aromatic compounds in pesticides to interact with the aromatic structures of biochar [65]. 

Biochar has emerged as a promising and sustainable method for removing pesticides from water bodies, demonstrating significant effectiveness in various studies. Its unique properties, such as high surface area and porosity, enhance its adsorption capabilities, making it a cost-effective solution for water remediation. Biochar derived from palm kernel shells modified with melamine achieved paraquat removal efficiencies of up to 99.7% in aqueous solutions [80]. Also, Cui et al. [81] showed that biochar exhibited an impressive adsorption capacity of 738.0 mg/g for neonicotinoid insecticides, with degradation efficiencies reaching 100%. In a study conducted by Yao et al. [65], KOH-activated biochar from crayfish shells demonstrated a maximum adsorption capacity of 40.41 mg/g for acetamiprid, utilizing multiple adsorption mechanisms. Biochar interacts with pesticides through various mechanisms, including π-π interactions, hydrogen bonding, and pore filling [65,81]. The combination of biochar with wetland plants enhances phytoremediation, reducing toxic effects and improving overall removal efficiency [80].

Kaur et al. [82] noted limitations like conventional methods struggle due to low concentrations and complex structures, and advanced methods can be expensive and produce secondary pollutants. Murtaza et al. [83] found that optimal pyrolysis conditions for enhanced biochar properties and further exploration of non-woody feedstock biochar applications in water treatment can serve as a further direction for biochar water remediation. Devenadad et al. [84] demonstrate that magnetic biochar, produced from waste packaging wood, effectively removes over 85% of chlorpyrifos from water. This innovative approach offers a cost-effective and sustainable solution for pesticide removal, addressing both waste management and environmental concerns. In another study by Koippully Manikandan et al. [57], rice husk biochar immobilized with chlorpyrifos-degrading Aeromonas veronii demonstrated significant efficacy in removing chlorpyrifos from water, achieving a 96.25% removal rate within 24 h, highlighting its potential as a cost-effective and sustainable pesticide remediation method. Zhou et al. [85] found that nitrogen-doped biochar enhances photocatalytic activity when coupled with Bi_2_WO_6_, demonstrating efficient adsorption and degradation of pesticides like chlorpyrifos, making it a cost-effective and sustainable method for water decontamination. BWO/N_3_-BC degrades 99.0% of chlorpyrifos in 0.5 h, and degradation rate constants are significantly higher than pure BWO and N_3_-BC.

Adsorption is the predominant mechanism through which biochar removes pesticides. This process involves the accumulation of pesticide molecules on the surface or within the porous structure of biochar [29]. Adsorption efficacy depends on several factors, including surface area, pore size distribution, functional groups, and the hydrophobicity of both biochar and pesticides [81]. Functional groups on biochar surfaces, such as carboxyl, hydroxyl, and carbonyl groups, can form chemical bonds or complexes with pesticides. This mechanism is particularly significant for polar pesticides or those containing functional groups capable of chemical interaction [86]. Also, pesticides and PAHs can be removed via partitioning, redox reactions, catalytic activity, microbial activity support, and pH modulation, but these mechanisms are not considered the main ones.

A comparative analysis of biochar and other adsorbents for PAH and pesticide removal is presented in Table 4. Although biochar has gained considerable attention due to its eco-friendliness, cost-effectiveness, and versatile functionality, it is important to evaluate performances alongside other adsorbents like activated carbon, zeolites, and advanced nanomaterials to understand its relative advantages and limitations.

While biochar offers a sustainable and cost-effective alternative to conventional adsorbents like activated carbon, its performance often requires enhancement through surface modifications. Compared to advanced materials like nanomaterials, biochar’s primary advantage lies in its low cost and environmental friendliness, although it may lack the same adsorption capacity. Addressing limitations such as regeneration challenges and variability in performance will be critical for scaling biochar’s application in water treatment systems.

Further research should focus on hybrid approaches that combine biochar with other materials to leverage the strengths of multiple adsorbents for enhanced efficiency in organic pollutant removal.

## 4. The Scalability and Long-Term Effectiveness of Biochar Uses for PAH and Pesticides Removal

The scalability and long-term effectiveness of biochar for PAH and pesticide remediation face several challenges, particularly in diverse environmental conditions. These challenges stem from the complex interactions between biochar properties and environmental factors, which can significantly affect its performance in contaminant removal. However, its scalability and long-term effectiveness remain topics of ongoing research and debate. These challenges largely stem from the complex interplay between biochar properties and environmental factors, which significantly influence its performance.

The performance of biochar in adsorbing PAHs and pesticides is also influenced by environmental conditions, including the following [49,75,89]: (1) pH: The pH of the solution can alter the surface charge of biochar and the ionization state of pesticides. For instance, acidic conditions may enhance the adsorption of basic pesticides by increasing electrostatic attraction; (2) coexisting organic matter: Natural organic matter (NOM) in water can compete with pollutants for adsorption sites on biochar, reducing efficiency. Alternatively, NOM can alter the pollutant’s solubility and biochar’s surface properties, indirectly affecting interactions; (3) temperature: higher temperatures may enhance the diffusion of pollutants into biochar pores but can also reduce adsorption due to desorption effects; and (4) optimizing these factors in real-world applications ensures maximum efficiency of biochar in water treatment.

The effectiveness of biochar varies significantly based on the biomass feedstock and pyrolysis conditions, which complicates large-scale production [90]. The type of biomass used to produce biochar affects its surface area, pore structure, and functional groups, which are critical for adsorption capacity [91]. Pyrolysis temperature and duration can alter biochar characteristics, impacting its efficacy in different soil types and contaminant profiles and excessive application of biochar can lead to negative environmental impacts, necessitating careful management strategies. Also, the economic feasibility of producing biochar at scale remains uncertain, impacting its widespread adoption [51]. All factors that affect the scalability and long-term effectiveness of biochar used for PAH and pesticide removal are summarized in Table 5.

Despite these challenges, ongoing research aims to optimize biochar production and application methods, potentially enhancing its effectiveness and sustainability in diverse environments. However, the complexity of environmental interactions and the need for tailored solutions remain significant hurdles in the widespread adoption of biochar for pesticide remediation. Despite these challenges, biochar’s potential for pollutant degradation remains promising, warranting further research to optimize its application and mitigate risks associated with its use. While biochar presents a sustainable approach to pesticide removal, challenges remain regarding the scalability and long-term effectiveness of biochar in diverse environmental conditions. Further research is needed to optimize its application in various water bodies, e.g., explore optimal biochar conditions for pesticide remediation and investigate biochar’s impact on emerging micro-pollutants. Regardless of whether it is modified or unmodified biochar, certain limiting factors are repeated in the literature, and there is limited information on the long-term effectiveness of biochar and a lack of standardized protocols for biochar application in wastewater treatment.

## 5. Summary and Conclusions

Using biochar for water remediation has gained significant attention in recent years due to its effectiveness in pollutant removal and its sustainability as a treatment material. Biochar, produced by pyrolyzing various biomass wastes, has a high specific surface area and a complex pore structure, making it ideal for adsorbing contaminants such as pesticides and organic pollutants. Studies show that biochar can achieve removal efficiencies above 90% for organic compounds when used in optimal conditions. Specifically, modified biochar, such as that combined with nano zero-valent iron (nZVI), enhances pollutant removal by facilitating adsorption and enabling redox reactions for persistent organic pollutants like PAHs, achieving removal rates up to 98% for specific pesticides and dyes in experimental setups.

Despite these promising results, the use of biochar in water remediation has limitations. Many studies report success in laboratory conditions, yet achieving similar effectiveness in real-world applications is challenging. Factors such as variability in water chemistry, the presence of competing ions, and fluctuating pH can affect biochar’s performance. Additionally, while modified biochar can significantly enhance removal rates, their synthesis often involves complex processes, such as chemical activation or nanomaterial integration, which may increase costs and environmental impact. Another limitation is biochar’s tendency for desorption under certain conditions, leading to potential re-release of contaminants into the environment. For instance, studies indicate that biochar’s adsorption efficiency for specific pollutants may decrease by over 30% in environments with high salinity or complex organic mixtures.

Current research has gaps in understanding the long-term stability and reusability of biochar-based remediation systems in natural settings. Future directions could explore biochar composites that are more resistant to variable environmental conditions, as well as cost-effective and environmentally friendly modifications. Additionally, investigating the interaction mechanisms at a molecular level using advanced techniques, such as density functional theory (DFT) calculations, may improve our understanding of biochar’s interaction with pollutants. Enhanced biodegradability and recovery methods for biochar are also recommended to support its sustainable use in larger-scale applications.

For instance, integrating biochar with AOPs or microbial treatments may offer synergistic effects, improving the removal of complex organic pollutants and enhancing degradation rates. Research on optimizing biochar’s surface properties through chemical and physical modifications could also provide insights into tailoring biochar for specific pollutants.

Biochar’s potential for integration into circular economy practices also presents a promising avenue. The use of waste-derived biochar not only provides a method for waste valorization but also supports sustainable water remediation practices. However, for biochar to be a feasible large-scale solution, further studies are needed to assess the environmental impacts of its production and disposal. Additionally, evaluating the economic feasibility and energy requirements of biochar production from different feedstocks is crucial for its commercialization.

## Figures and Tables

**Table 4:** Comparison of biochar and other available sorbents.

Adsorbent	Advantages	Disadvantages	References
Biochar	Sustainable, cost-effective, tunable surface properties, and versatile applications	Lower initial performance, variability in quality, and regeneration challenges	Qui et al. [61] Jha et al. [87] Srivatsav et al. [88]
Activated Carbon	High adsorption capacity, widely available, and well researched	High cost, environmental impact, and fouling issues	
Zeolites	Selective adsorption and thermal stability	High cost and limited effectiveness for hydrophobic pollutants	
Nanomaterials	Superior adsorption efficiency and customizable properties	Expensive, scalability issues, and potential toxicity	

**Table 5:** Factors that affect on scalability and long-term effectiveness of biochar.

Factor	Explanation of Why This Is Important	References
Biochar properties affecting removal efficiency: surface area and porosity; chemical functional groups; surface charge; hydrophobicity and polarity	The performance of biochar in contaminant removal depends heavily on its intrinsic properties, which are shaped by the feedstock type, pyrolysis conditions, and post-production modifications.	Kong et al. [60] Dong et al. [29]
Environmental factors influencing biochar efficiency: pH of the environment; presence of natural organic matter (NOM); coexisting ions; temperature and humidity	Biochar’s performance in contaminant removal is also highly sensitive to environmental conditions, which can either enhance or hinder its efficacy.	Lechmann and Joseph [92]
Challenges in scalability: feedstock variability; environmental variability; regeneration and reusability; cost-effectiveness	Scaling up biochar-based water treatment systems for real-world applications introduces additional complexities:	Fdez-Sanromán et al. [93] Al Rumaihi et al. [94]
Strategies to enhance scalability and long-term effectiveness; tailored design; multi-functionality; adaptation to environmental conditions, life-cycle management	Addressing the challenges associated with biochar’s scalability and long-term effectiveness requires innovative approaches:	Ibitoye et al. [95] Guo et al. [96]
